# Supplementary material for: Landscape of Clinical Trials in Cancer Cachexia: Assessment of Trends From 1995–2024
Source: medRxiv. 2025 Jun 25:2025.03.14.25323917. Preprint. [Version 3] doi: 10.1101/2025.03.14.25323917 (PMC11952627; doi:10.1101/2025.03.14.25323917)
Supplement: 1 — Supplemental File 1. List of clinical trials included in this review. Identification number is the NCT code under which the clinical trial is registered in ClinicalTrials.gov. The status is completed with (w/) results, completed without (w/o) results, still active, or withdrawn (terminated) before completion. Supplemental File 2. Summary of search results by terms and status of the clinical trials. Supplemental File 3. This table list the reasons for withdrawn or anticipated termination before completion of clinical trials studying cancer cachexia registered in ClinicalTrials.gov. Supplemental File 4. Phases and status of clinical trials exploring cancer cachexia effects on ClinicalTrials.gov in Phase1-4 or No Phase. Supplemental File 5. Clinical trials registered on ClinicalTrials.gov that used Fearon’s definition or any other criteria to diagnose cancer cachexia within each status. Supplemental File 6. Clinical trials stratified by clinical trial status studying cachexia but classified under other terms different than “cachexia”. Supplemental File 7. A) Study population of clinical trials registered on ClinicalTrials.gov investigating cancer cachexia. B) Population dropout percentage within each clinical trial. Supplemental File 8. Cancer type and cancer stage of clinical trials registered on ClinicalTrials.gov investigating cancer cachexia. Supplemental File 9. Clinical trials approach and intervention utilized: drug testing, dietary supplement, exercise/physical therapy, a combination of 2 or more interventions (multimodal), others, or none or non-stated intervention of clinical trials registered on ClinicalTrials.gov investigating cancer cachexia. Supplemental File 10. Pharmaceutical drugs and dietary supplements evaluated in CTx between 1995–2024. Supplemental File 11. Biomarkers measured in cancer cachexia clinical trials registered on ClinicalTrials.gov. Supplemental File 12. Outcome measures of cachexia in the clinical trials registered on ClinicalTrials.go [file NIHPP2025.03.14.25323917V3-supplement-1.pdf]

Supplemental File 1 - List of clinical trials

Identification Number	Status	Study Title
NCT00467844	Completed w/ Results	Double-Blind, Placebo-Controlled, Dose-Finding Study of the Effect of GTx-024 on Muscle Wasting (Cachexia) in Patients with Cancer
NCT00815685	Completed w/ Results	A Pilot Study of Eicosapentaenoic Acid (EPA) in Patients with Cancer Cachexia
NCT01433263	Completed w/ Results	A Randomized, Double-blind, Placebo-controlled Multi-center Study of BYM338 for Treatment of Cachexia in Patients with Stage IV Non-small Cell Lung Cancer or Stage III/IV Adenocarcinoma of the Pancreas
NCT00527319	Completed w/ Results	A Pilot Open Label Randomized Controlled Study to Evaluate the Dose Tolerance Safety and Efficacy of VT-122 Regimen for the Treatment of Cachexia in Subjects with Stage IV Non-Small Cell Lung Cancer
NCT02968979	Completed w/ Results	Cross-sectional, Multicentric, Non-interventional Study to Assess the Frequency and the Management of Cachexia and Associated Symptoms in Patients with Non-small Cell Lung Cancer
NCT00878995	Completed w/ Results	Nutrition and Anabolic Interventions in Squamous Cell Carcinoma
NCT01395914	Completed w/ Results	Anamorelin HCl in the Treatment of Non-Small Cell Lung Cancer-Cachexia (NSCLC-C): An Extension Study
NCT01387282	Completed w/ Results	Anamorelin HCl in the Treatment of Non-Small Cell Lung Cancer-Cachexia (NSCLC-C): A Randomized Double-Blind Placebo-Controlled Multicenter Phase III Study to Evaluate the Safety and Efficacy of Anamorelin HCl in Patients With NSCLC-C
NCT01387269	Completed w/ Results	Anamorelin HCl in the Treatment of Non-Small Cell Lung Cancer-Cachexia (NSCLC-C): A Randomized Double-Blind Placebo-Controlled Multicenter Phase III Study to Evaluate the Safety and Efficacy of Anamorelin HCl in Patients With NSCLC-C
NCT01355497	Completed w/ Results	Phase III, Randomized, Double-Blind, Placebo-Controlled Study of the Effect of GTx-024 on Muscle Wasting in Patients with Non-Small Cell Lung Cancer on First Line Platinum Plus a Non-Taxane Chemotherapy
NCT01355484	Completed w/ Results	Phase III, Randomized, Double-Blind, Placebo Controlled Study of the Effect of GTx-024 on Muscle Wasting in Patients with Non-Small Cell Lung Cancer on First Line Platinum Plus a Taxane Chemotherapy

… Supplemental File 1 Continue

Identification Number	Status	Study Title
NCT00513357	Completed w/ Results	A Randomized Clinical Trial of Melatonin Versus Placebo and the Effect on Appetite in Advanced Cancer Patients
NCT04299048	Completed w/ Results	A Phase 1B, 12-Week, Open-label Study to Assess the Safety, Tolerability, Pharmacokinetics and Pharmacodynamics following repeated subcutaneos administration of PF-06946860 in Patients with Cancer and Cachexia
NCT00040885	Completed w/ Results	Docetaxel and Infliximab/Placebo In Non-Small Cell Lung Cancer (NSCLC) Patients Greater Than Or Equal To 65 Years Of Age Or In NSCLC Patients With Poor Performance Status: A Double-Blind Randomized, Placebo-Controlled Trial To Prevent And Treat Wasting, Anorexia, And Asthenia In Chemotherapy-Naive And Previously-Treated Patients
NCT03524755	Completed w/ Results	Effects of a Progressive Resistance Training in Cachectic Cancer Patients During Radiotherapy - a Randomized Controlled Pilot Trial
NCT02138422	Completed w/ Results	A Double Blind, Placebo Controlled Pivotal Phase III Study Evaluating Xilonix™ in Symptomatic Colorectal Cancer Patients Refractory to Standard Therapy
NCT00081250	Completed w/ Results	Phase III Double-Blind, Placebo-Controlled Randomized Comparison of Creatine for Cancer-Associated Weight Loss
NCT02359123	Completed w/ Results	Cannabics Capsules as Treatment to Improve Cancer Related Cachexia and Anorexia Syndrome in Advanced Cancer Patients- Pilot Study
NCT03254173	Completed w/ Results	Mirtazapine for Treatment of Cancer Associated Anorexia-cachexia: A Randomized Controlled Clinical Trial
NCT02400398	Completed w/ Results	A Longitudinal, Single Institution Study Evaluating Weight Stability in Advanced Pancreatic Cancer Patients with Cachexia Who Are Receiving Enteral Feeding
NCT03207724	Completed w/ Results	A Phase I Study of Onivyde and 5-FU in Combination with Xilonix for Advanced Pancreatic Cancer with Cachexia

… Supplemental File 1 Continue		
**Identification Number**	**Status**	**Study Title**
NCT04208334	Completed w/ Results	A Double-blind, Placebo-controlled Randomized Trial Phase II Evaluating the Effect of Curcumin for Treatment of Cancer Anorexia-Cachexia Syndrome in Patients with Stage III-IV of Head and Neck Cancer
NCT03283488	Completed w/ Results	Randomized, Double-blind Clinical Trial of the Use of Mirtazapine Versus Megestrol for the Control of Anorexia-cachexia in Cancer Patients in Palliative Care.
NCT04585841	Completed w/ Results	The Effect of Medical Cannabidiol on Lean Body Mass in Patients Receiving Oxaliplatin or Paclitaxel Based Chemotherapy
NCT00031707	Completed w/ Results	Phase III Double-Blind, Placebo-Controlled Randomized Comparison of Megestrol Acetate (Megace) Versus an N-3 Fatty Acid (EPA) Enriched Nutritional Supplement Versus Both for the Treatment of Cancer Cachexia and Anorexia
NCT00919659	Completed w/ Results	Parenteral Nutrition Support for Patients with Pancreatic Cancer. A Phase II Study.
NCT00329615	Completed w/ Results	Insulin Treatment in Cancer Cachexia: Effects on Survival, Metabolism and Physical Functioning. A Randomized Prospective Study.
NCT00219817	Completed w/ Results	Safety and Efficacy of RC-1291 HCl in Patients with Cancer Related Anorexia and Weight Loss
NCT00267358	Completed w/ Results	Placebo Controlled, Randomized Safety and Efficacy Study of RC-1291 in Cancer Anorexia/Cachexia.
NCT00066248	Completed w/ Results	The Effect of Cyproheptadine Hydrochloride (Periactin) and Megestrol Acetate (Megace) on Weight in Children with Cancer/Treatment Related Cachexia
NCT00046904	Completed w/ Results	Phase III Placebo-Controlled, Randomized, Double-Blind Comparison of Etanercept (Enbrel) Versus Placebo for the Treatment of Cancer-Associated Weight Loss and Anorexia
NCT00053053	Completed w/ Results	A Double-Blind Study of Nutritional Intervention for The Treatment of Cancer Cachexia Using Juven® Nutritional Supplement
NCT00933361	Completed w/ Results	Individual Dose-escalated Bi-daily sc Ghrelin in Cancer Cachexia: A Phase I/II Study

… Supplemental File 1 Continue

Identification Number	Status	Study Title
NCT00477919	Completed w/ Results	E-MOSAIC: A Multicenter Randomized Controlled Phase III Study of Longitudinal Electronic Monitoring of Symptoms and Syndromes Associated with Advanced Cancer in Patients Receiving Anticancer Treatment in Palliative Intention
NCT00895726	Completed w/ Results	Open-Label, Pilot Phase I/II Study of the Efficacy and Safety of APD209 in Patients with Cachexia and Advanced Malignancy
NCT00489593	Completed w/ Results	Phase I Dose-Finding Pilot Study of the Safety and Tolerability of Olanzapine in Patients with Advanced Cancer and Weight Loss
NCT01596933	Completed w/ Results	Multi-centric, Placebo-controlled Trial of Omega-3 Fatty Acid Supplementation in Weight Maintenance and Preservation of Lean Body Mass, in (Pre)Cachectic Head and Neck Cancer Patients, Undergoing Curative Radio (Chemo)Therapy
NCT02350855	Completed w/ Results	To Study the Impact of Nutritional Intervention in Delaying the Progression of Cachexia to Refractory Cachexia in Female Cancer Patients: A Study Based in India
NCT01419145	Completed w/ Results	A Feasibility Study of Multimodal Exercise/Nutrition/Anti-inflammatory Treatment for Cachexia - the Pre-MENAC Study
NCT01622036	Completed w/ Results	The PreMiO Study: The Prevalence of Malnutrition in Oncology.
NCT01604642	Completed w/ Results	Role of Activin A (ActA) in the Human Cancer Cachexia
NCT02561143	Completed w/ Results	To Evaluate the Effectiveness of Nutritional Counselling and "Improved Atta" Supplementation in Delaying Progression of Cachexia to Refractory Cachexia in Adult Cancer Patients in Indian Population
NCT02148159	Completed w/ Results	Impact of Mechanism Based Acupuncture Intervention to Improve Weight Loss in GI Cancer Patients with Cachexia
NCT02515032	Completed w/ Results	A 12-week Double Blind, Placebo Controlled, Randomised Pilot Study Assessing Safety and Tolerability of Nutrifriend Cachexia in Patients Diagnosed with Non-Small Cell Lung Cancer (NSCLC) With Involuntary Weight Loss
NCT02293239	Completed w/ Results	Effects of Whole-Body Electromyostimulation (WB-EMS) Combined with Individualized Nutritional Support on Patients with Malignant Disease Undergoing Curative or Palliative Anti-cancer Treatment

… Supplemental File 1 Continue

Identification Number	Status	Study Title
NCT01614990	Completed w/ Results	Pilot Clinical Trial of Repeated Doses of Macimorelin to Assess Safety and Efficacy in Patients with Cancer Cachexia
NCT05837741	Completed w/ Results	Standard Hypercaloric, Hyperproteic vs Leucine-enriched Oral Supplements in Patients with Cancer-induced Sarcopenia
NCT02877368	Completed w/ Results	Sarcopenia in Patients with Gastrointestinal Stromal Tumours
NCT02779868	Completed w/ Results	Effect of Omega-3 Supplementation on Body Composition, Functional Capacity, Inflammatory Profile and Quality of Life in Cervix Cancer Patients Undergoing Chemoradiotherapy
NCT01484821	Completed w/ Results	Assessment of in Vivo Skeletal Muscle Viscoelasticity (ARFI Imaging) With Aging and Cancer (CAPARFI)
NCT04714203	Completed w/ Results	Assessment of the Prevalence of Sarcopenia in Early Palliative Cancer Patients
NCT01564693	Completed w/ Results	Neuroimaging of Hypothalamic Activity During Cancer Anorexia
NCT04153019	Completed w/o Results	psycho-educational and Rehabilitative Intervention for the Oncological Patient with Cachexia and his Caregiver: A Feasibility Study
NCT02199912	Completed w/o Results	ANC2 Pilot Study of the Relationship Between Clinical and Paraclinical Markers During Situations of Cachexia and Pre-cachexia in Patients Over 70 Years with Colorectal Surgery
NCT03583177	Completed w/o Results	Study of Muscle Wasting Mechanisms and Biomarkers in Hemodialysis Patient
NCT03144128	Completed w/o Results	The Contribution of Vitamin D to Muscle Metabolic Function in Cancer Cachexia
NCT04802486	Completed w/o Results	Evaluating the Effects of a 12 Week Home-based Exercise Intervention on Physical Performance in Patients with Cancers of the Gastrointestinal Tract with Pre- Cachexia or Cachexia: A Pilot Study
NCT02983500	Completed w/o Results	Tablet-based Prospective Patient Reported Outcomes (PRO) Registry in Patients with Advanced NSCLC or Advanced Pancreatic Cancer with Focus on Cancer Cachexia

… Supplemental File 1 Continue

Identification Number	Status	Study Title
NCT03012139	Completed w/o Results	Cancer-Associated Muscle Atrophy and Weakness: An Investigation of Etiology
NCT04643613	Completed w/o Results	Role of Ready to Drink Nutritional Formula (Protison Enriched With ω-3 FA and BCAA) Supplemented by Nasogastric Tube Feeding in Various Cancer Patients: A Randomized Clinical Trial
NCT03958032	Completed w/o Results	Effects of Sarcopenia on Early Postoperative Outcomes in Patients Undergoing Surgical Treatment for Gastric Cancer
NCT03881826	Completed w/o Results	Investigation of the Gut Microbiota in Patients with Acute Myeloid Leukemia
NCT05023499	Completed w/o Results	Perioperative Glutamine Supplementation Restores Atrophy of Psoas Muscle in Gastric Adenocarcinoma Patients Undergoing Gastrectomy
NCT04131426	Completed w/o Results	Evaluating the Effects of Dietary Supplementation with Remune on Cancer Associated Weight and Muscle Loss with and Without Exercise: A Randomized Pilot Feasibility Study.
NCT03743051	Completed w/ Results	A Phase 3 Randomized, Double-Blind, Placebo-Controlled, Multicenter Study to Evaluate the Efficacy and Safety of Anamorelin HCl for the Treatment of Malignancy Associated Weight Loss and Anorexia in Adult Patients with Advanced Non-Small Cell Lung Cancer (NSCLC)
NCT00014248	Completed w/o Results	A Phase I Study and Pharmacokinetics of Adenosine 5'- Triphosphate (ATP) When Administered by Intravenous Infusion on A Multiple Weekly Dose Schedule To Patients With Advanced Malignancies (Solid Tumors)
NCT00004912	Completed w/o Results	Phase II Trial of Progressive Resistance Training with Megestrol Acetate for the Treatment of Cancer-Related Weight Loss
NCT00378131	Completed w/o Results	A 12-Week, Double-Blind, Randomized, Placebo-Controlled, Parallel Group Phase II Study Comparing the Safety, Tolerability and Beneficial Effects of Daily Doses of RC-1291 and Placebo in Patients with Cancer Anorexia/Cachexia

… Supplemental File 1 Continue

Identification Number	Status	Study Title
NCT00094562	Completed w/o Results	AAFA™ Fish Oil Nutritional Supplementation to Maintain Body Weight in Patients with Disease-Related Weight Loss
NCT00486304	Completed w/o Results	Determine the safety of the antioxidant-deficient diet (ADD) in controlling cachexia in patients with oropharyngeal cancer receiving chemoradiotherapy.
NCT00899158	Completed w/o Results	Role of Caspase-3, Phosphatidylinositol-3 Kinase (PI3K), and 3-methylhistidine (3-MH) in the Pathophysiology of Skeletal Muscle Loss in Weight-losing Pancreas Cancer Patients
NCT00866970	Completed w/o Results	A Phase II Study to Determine the Safety, Efficacy, and Pharmacokinetics of Multiple Intravenous Doses of ALD518 80 mg, 160 mg, and 320 mg Versus Placebo Administered to Patients with Non-Small Cell Lung Cancer-Related Fatigue and Cachexia
NCT01136083	Completed w/o Results	Effect of Exercise Training on Angiogenesis and Cachexia in Lung Cancer Patients
NCT00994669	Completed w/o Results	Development of a Non-invasive Assessment of Skeletal Muscle Loss in Cancer Patients
NCT01127386	Completed w/o Results	Effect of Lenalidomide (Revlimid®) in Solid Tumour Patients with Inflammatory Cancer Cachexia Syndrome on Lean Body Mass and Muscle Strength: A Multicenter, Proof-of-concept Study of Fixed Dose or CRP-response-guided Dose of Lenalidomide in Relation to New Standard Basic Cachexia Management (Receiving Placebo).
NCT01939340	Completed w/o Results	Prevalence and Impact of Cachexia in Cancer Patients Undergoing Chemotherapy
NCT01238107	Completed w/o Results	A Multicentre, Randomised, Double-blind, Placebo-controlled, Phase II Clinical Study to Evaluate Doses of MT-102 in Subjects with Cachexia Related to Stage III and IV Non-small Cell Lung Cancer and Colorectal Cancer

… Supplemental File 1 Continue

Identification Number	Status	Study Title
NCT01533909	Completed w/o Results	Development of a Screening Tool to Detect and Stage Cachectic Cancer Patients
NCT01694602	Completed w/o Results	Non-Invasive Assessment of Skeletal Muscle Loss in Cancer Patients - Phase 2
NCT04699760	Completed w/o Results	Single-blinded, Randomized, Controlled Trial About the Effects of n-3 LCPUFAs on Weight and Functional Status in Patients with Colorectal Cancer.
NCT03162042	Completed w/o Results	Role of Myostatin, Activin A and Follistatin Cachexia of ENT Cancers
NCT03263520	Completed w/o Results	Impact of the Use of Nandrolone on the Treatment of Malnutrition Induced by Cancer
NCT05264038	Completed w/o Results	A Phase 1, Randomized, Double-Blind, Dose-Ranging, Placebo-controlled Study to Evaluate the Safety, Pharmacokinetics, and Pharmacodynamics Effects of OC514 in Healthy Adult Volunteers
NCT05323604	Completed w/o Results	Impact of Sarcopenia on Post-operative Course for Cancer Patients Operated of Colorectal Surgery Using a Simple Measurement Method of the Psoas Muscle Surface Area
NCT05583292	Completed w/o Results	The Effectiveness of ASA, SORT and Sarcopenia Indices in Estimating Mortality and Morbidity in Gastrointestinal Tumour Surgery
NCT03032224	Completed w/o Results	Analysis of Sarcopenia and Body Composition in Relation to Outcome in a Cohort of Patients with Esophageal Cancer or Cancer of the Gastroesophageal Junction Before and After Surgery with Curative Intent
NCT03915288	Completed w/o Results	Moderate Continuous Training Versus HIIT on Cardiometabolic and Psychosocial Variables in Cancer Stadium II. Control Randomized Trial
NCT03770117	Completed w/o Results	A Pilot Study of the Effect of Prehabilitation on Markers of Sarcopenia in Patients Undergoing Pancreatoduodenectomy for Malignant Disease

… Supplemental File 1 Continue

Identification Number	Status	Study Title
NCT03962140	Completed w/o Results	Computed Tomography Assessment of Body Fat Distribution in Endometrial Cancer Patients
NCT04815551	Completed w/o Results	A Phase 1, First-in-human, Randomized, Placebo Controlled, Double Blind, Single Ascending Dose (SAD) Study to Evaluate the Safety, Tolerability, Pharmacokinetics, Pharmacodynamics and Immunogenicity of AV-380 in Healthy Subjects
NCT03960034	Completed w/o Results	Cachexia in Advanced NSCLC Patients: Diagnosis, Characterization, Prognosis, Functional Implications and Validation of Skeletal Muscle Disfunction Markers
NCT03058107	Completed w/o Results	Tight Caloric Control in the Cachectic Oncologic Patient (TiCaCONCO or CoCooN) Can Stabilize Body Weight, Thereby Reducing Morbidity and Mortality: Validation of the Pilot Study - A Randomized Controlled Double-Blind Trial
NCT04803305	Completed w/o Results	A 6-Week, Randomized, Double-blind, Sponsor-open Study to Assess the Effect of Repeated Sucutaneous Administration of PF-06946860 on Appetite in Participants with Advanced Cancer and Cachexia, Followed by an 18-week Open-Label Treatment Period
NCT02330926	Completed w/o Results	A Randomised, Open-label Trial of a Multimodal Intervention (Exercise, Nutrition and Antiinflammatory Medication) Plus Standard Care Versus Standard Care Alone to Prevent/Attenuate Cachexia in Advanced Cancer Patients Undergoing Chemotherapy
NCT00060502	Completed w/o Results	A Phase II, Multicenter, Randomized, Double-Blind, Placebo Controlled Study Evaluating the Efficacy and Safety of Anti-TNF a Monoclonal Antibody (Infliximab) to Treat Cancer-Related Cachexia in Subjects with Pancreatic Cancer.
NCT04161794	Completed w/o Results	Multimodal Intervention (Dietary Counselling, Fish Oil and Physical Training) for Patients with Non-small Cell Lung Cancer, a Feasibility Study
NCT02500004	Completed w/o Results	Brown Adipose Tissue Activity and Energy Metabolism in Cachexia Induced by Cancer or Chronic Disease

… Supplemental File 1 Continue		
**Identification Number**	**Status**	**Study Title**
NCT00196885	Completed w/o Results	Effect of N-Acetylcysteine on Skeletal Muscle in Cachectic Cancer Patients Undergoing a Resistance Training Program (Phase 2 Study)
NCT03743064	Completed w/o Results	A Phase 3 Randomized, Double-Blind, Placebo-Controlled, Multicenter Study to Evaluate the Efficacy and Safety of Anamorelin HCl for the Treatment of Malignancy Associated Weight Loss and Anorexia in Adult Patients with Advanced Non-Small Cell Lung Cancer (NSCLC)
NCT03316157	Completed w/o Results	Exercise and Nutritional Rehabilitation in Patients with Cancer, Randomised (1:1) Unblinded Feasibility Trial of a Rehabilitation Programme (Exercise and Nutrition) Versus Waiting List Control, in Patients with Advanced Cancer
NCT00003077	Completed w/o Results	Phase I/II Trial of Omega-3 Fatty Acids for Cancer Cachexia
NCT03200639	Completed w/o Results	Comparison of High Intensity Interval Body Weight Training Versus Combined Training on Body Composition, Physical Function, Metabolic Risk and Inflammation in Postmenopausal Women with and Without Gynecological Cancer
NCT02788955	Completed w/o Results	Decreasing the Burden of Sarcopenia in Cancer Through Targeted Nutrition Intervention: A Feasibility Study
NCT06093009	Completed w/o Results	The Significance of Postoperative Muscle Wasting in Pancreatic Cancer
NCT02107664	Completed w/o Results	The Palliative Radiotherapy and Inflammation Study: Palliative Radiotherapy of Bone Metastasis. Classification, Inflammatory Biomarkers, and Longitudinal Clinical Follow-up
NCT03042442	Completed w/o Results	Relation Between Cachexia, Diabetes and periNeural Invasion in PANcreatic Cancer- Biomarkers Substudy
NCT05623852	Active	A Phase II Trial for Oligo-Fucoidan in Cancer Cachexia and Sarcopenia
NCT05376592	Active	Association of Genomic Polymorphisms with Cancer Cachexia in Subjects with Pancreatic Adenocarcinoma
NCT05865535	Active	A Phase 1 Dose Escalation Study of AV-380 in Combination with Standard of Care Chemotherapy in Metastatic Cancer Patients with Cachexia and Elevated GDF-15 Levels

… Supplemental File 1 Continue

Identification Number	Status	Study Title
NCT05912465	Active	The Role of 18F-FDG PET/CT in the Early Prediction of Cachexia in Lung Cancer Patients
NCT04127981	Active	A Pilot Bioimaging Trial of Cancer Cachexia
NCT05128318	Active	Study of the Tumor-adipose Tissue Dialogue: Role of Tumor Acidosis in the Induction of Adipocyte Lipolysis
NCT05731076	Active	Efficacy of Self-Management Support on Nutritional Status in Lung Cancer Patients with Cancer Cachexia Syndrome: A Randomized Clinical Trail
NCT06073431	Active	Longitudinal Observational Trial to Uncover Subtypes of Cancer Cachexia
NCT05275075	Active	Pilot Trial to Identify microRNAs in Cachexia in Patients with Pancreatic Carcinoma
NCT05915325	Active	Physical Training for Elderly Cancer Patients with Cachexia (TEECH-01): A Prospective Clinical Trial
NCT04627376	Active	Effectiveness of a Multimodal Education and Support Program for the Prevention of Cancer Related Cachexia for Patients and Their Family Caregiver
NCT05606523	Active	Can Fecal Microbiota Transplantation of Cachectic Patients with Pancreas Cancer Impair Body Weight Gain in Germ-free Mice? The EXTRA Study
NCT04906746	Active	Pilot Study of Ruxolitinib in the Treatment of Cancer Cachexia
NCT04864431	Active	The Effect of Vitamin D on Factors Contributing Pre-Cachexia and Cachexia, a Study on Epithelial Ovarian Cancer
NCT05028192	Active	Mitochondria Preservation by Exercise Training: A Targeted Therapy for Cancer and Chemotherapy-induced Cachexia.
NCT04600154	Active	Effects of MS-20 on Gut Microbiota and Risk/Severity of Cachexia in Patients Receiving Chemotherapy for Pancreatic Cancer
NCT04469504	Active	Prehabilitaion Program for Sarcopenic Patients Prior to Pancreaticoduodenectomy for Periampullary Malignant Tumors

… Supplemental File 1 Continue

Identification Number	Status	Study Title
NCT06015971	Active	Omega-3 Enriched Supplements in the Nutritional Support of Patients with Gastrointestinal Cancer
NCT05294380	Active	Determination of Sarcopenia Risk and Related Factors in Pediatric Oncological Patients
NCT03641118	Active	Assessment of Measures of Sarcopenia and Whole-Body Physiology in Cancer Patients and Their Relationship to Surgical Outcome and Survival
NCT05833321	Active	Prospective Monocentric Study of the Detection of Sarcopenia in Clinical Practice in Patients with Ovarian or Endometrial Cancer Requiring Systemic Oncological Treatment
NCT05658263	Active	Bacterial Intestinal Gut Modification Around Cancer Surgery (BIG MACS) Diet
NCT02978521	Active	Effect of a Pulmonary Rehabilitation Program on Skeletal Muscle Mass, Pulmonary Function, Inflammatory Response and Overall Survival on Patients Diagnosed with Non-small-cell Advanced Cancer
NCT06203301	Active	The Relationships of Resistance Exercise, Walking, Myokine Secretion, Sarcopenia, Muscle Loss, Quality of Life, and Predictors in Cancer Patients Receiving Chemoradiotheray
NCT03256201	Active	Preoperative Exercise and Nutrition to Improve Pancreatic Cancer Outcomes by Targeting Sarcopenia: A Translational Pilot RCT
NCT05307367	Active	Identifying Molecular Factors Contributing to Cancer-associated Muscle Mass Loss and Providing Clinical Evidence for Exercise Mechanisms to Functionally Restore Muscle in Cancer
NCT06027242	Active	Oral Supplementation of Glutamine on Gastric Cancer Patients After Gastrectomy
NCT05778136	Active	Exercise in Lung Cancer Patients - Specific Training, Muscle Mass and Force Development
NCT05650827	Active	Feasibility and Effect of Resistance Training and Protein Supplementation in Patients with Advanced Gastroesophageal Cancer

… Supplemental File 1 Continue

Identification Number	Status	Study Title
NCT04899882	Active	Physical Fitness Impact of Early Physiotherapy Intervention with a Standardized Exercise Therapy Program in Adult Patients Receiving Intensive Induction Chemotherapy for Treatment of Acute Leukemia During Extended Hospitalization. Randomized Controlled Superiority Trial in Parallel Arms, Multicentric (KinHémo)
NCT05556239	Active	The Effect of Resistance Training in Patients with Malignant Lymphoma Undergoing Chemotherapy Treatment - the STAY STRONG TRIAL - a Randomized Controlled Trial.
NCT05899205	Active	Patient Recorded Indexing Measurements
NCT05257135	Active	Development of a Prospective Clinico-biological Database in Cachexia in Patients with Colon Cancer
NCT06250686	Active	Exercise and Nutrition Intervention in Ovarian Cancer - Development of a Care Concept and Evaluation in Routine Clinical Practice
NCT05642819	Active	Routine Evaluation of People Living with Cancer - Surgery
NCT05919147	Active	A Phase II, Randomized, Double-Blind, Placebo-Controlled Study to Investigate the Neuroendocrine Effects of Pioglitazone in Patients with Advanced Non-Small-Cell Lung Cancer and Cachexia
NCT05336266	Active	IIT2021-16-Hendifar-KetoROCX: A Feasibility Study of Ketorolac Treatment for Cachexia in Patients with Advanced Pancreatic Ductal Adenocarcinoma
NCT04098237	Active	Pancreatic-enzyme Replacement Therapy with Pancreaze (Pancrelipase) Delayed-release in Addition to Standard of Care for Borderline Resectable, Locally Advanced, and Advanced Pancreatic Adenocarcinoma Patients (PANCAX-3) With Cachexia and Exocrine Pancreatic Insufficiency
NCT04844970	Active	A Randomized, Double-blind, and Placebo Controlled Multicenter Phase II Trial Evaluating Anamorelin in the Prevention of Cancer Induced-Weight Loss and Anorexia in Patients Receiving First-line Treatment of Advanced Pancreatic Cancer

… Supplemental File 1 Continue

Identification Number	Status	Study Title
NCT06207708	Active	Role of Baseline Sarcopenia in Determining Acute Toxicity for Head and Neck Cancer Patients Treated with Curative Hadrontherapy: A Prospective Monoinstitutional Study
NCT05194397	Active	Intensive Tailored Exercise Training With NAD+ Precursor Supplementation to Improve Muscle Mass and Fitness in Adolescent and Young Adult Hematopoietic Cell Transplant Survivors
NCT03982082	Active	Feasibility Study of Ultrasound to Evaluate Muscle-Glycogen Content in Patients with Cancer
NCT03851133	Active	The Florida Pancreas Collaborative Next-Generation Biobank: Reducing Health Disparities and Improving Survival for Pancreatic Cancer
NCT04090619	Active	Frequency of Cachexia in Ambulatory Cancer Patients and Psychological Burden in Both Patients and Their Primary Caregivers Who Are Referred to an Outpatient Supportive Care Clinic
NCT05546476	Active	A Phase 2, Randomized, Double-blind, Placebo-controlled Study to Investigate the Efficacy, Safety and Tolerability of Ponsegromab in Patients with Cancer, Cachexia, and Elevated concentrations of GDF-15, Followed by an Optional Open-Label Treatment Period (PROACC -1)
NCT03637816	Active	Qualitative Study on Experiences Related to Anorexia and the Effects of Anamorelin and Placebo in Advanced Non-Small-Cell Lung Cancer Patients with Anorexia-Cachexia: A Preliminary Study
NCT02567669	Active	Muscle Loss and Fatigue as a Consequence of Radiotherapy
NCT04065984	Active	BiCyCLE: Neuro-Muscular Electrical Stimulation (NMES) and Its Effect on Changes in Body Composition Following Surgery for Locally Advanced Rectal Cancer - a Single Centre Double Blind Randomised Controlled Phase II Clinical Trial

… Supplemental File 1 Continue		
**Identification Number**	**Status**	**Study Title**
NCT03606317	Active	Retrospective Review of Cachexia in Lung and Gastrointestinal Cancer Patients
NCT03568019	Active	PET Avidity in Cachexia-Inducing Lung and Gastrointestinal Tumors
NCT04090619	Active	Frequency of Cachexia in Ambulatory Cancer Patients and Psychological Burden in Both Patients and Their Primary Caregivers Who Are Referred to an Outpatient Supportive Care Clinic
NCT06166134	Active	Radiological Evaluation of Sarcopenia in Patient Received Chemotherapy
NCT05771207	Active	Effects of Soy Protein and Whey Protein on Muscle, Gut Microbiota and Clinical Outcome in Patients with Lung Cancer-related Sarcopenia（LCRS）: A Randomized, Double-blind, Controlled Clinical Study
NCT05710809	Active	The SaVe Project-Sarcopenia and Vertigo in Aging Patients with Colorectal Cancer
NCT06137508	Active	Cancer-associated Cachexia in Patients with Incurable Gastroesophageal Cancer
NCT06077734	Active	In Vitro Quality Assessment of Myogenic Stem Cells in Multiple Patient Groups with Confirmed Skeletal Muscle Atrophy to Study Their Potential for Autologous Stem Cell Therapy
NCT06007794	Active	Correlation Between Ultrasound-assessed Quadriceps Muscle Mass and Baseline Whole-body Densitometry Muscle Index in the Post-cancer Population (JUMP Research II)
NCT05954117	Active	Effect of Chemotherapy on Metabolic Flexibility in the Context of Cachexia in Cancers of the Esophagus and Gastroesophageal Junction
NCT05856500	Active	A Prospective Open Controlled Study of Creatine Combined with Curcumin in the Intervention of Early Cachexia in Upper Gastrointestinal Tumors

… Supplemental File 1 Continue

**Identification Number**	**Status**	**Study Title**
NCT05420259	Active	Influence of a Combined Dietary and Exercise Intervention on Cytokine Profile and NK Cell Activation in Cancer Patients Undergoing Neoadjuvant Treatment.
NCT06277986	Active	Clinical Value of Tumor Cell-derived Exosomal miRNA in Early Diagnosis of Gastric CancerCachexia
NCT03191955	Active	Biochemical and Functional Biomarkers of Cachexia in Cancer Patients
NCT00637728	Terminated	A Randomized, Double-blind, Placebo-controlled Study of Megestrol Acetate Concentrated Suspension for the Treatment of Cancer-associated Anorexia in Subjects with Lung or Pancreatic Cancer
NCT02515513	Terminated	The Role of Muscle Cachexia in Pancreatic Cancer
NCT04034745	Terminated	IIT2018-26 -Hendifar-NETCx: A Descriptive, Multicenter, Single-arm, Open Label Study to Analyze the Effect of Telotristat Ethyl on Weight Regulation/Gain
NCT02877966	Terminated	Evaluation of the Effects of a Stoichiometric Mixture of Amino Acids (Amixea) on Lean Body Mass and Muscle Strengh of Patients with Unresectable Advanced Non-Small Cell Lung Cancer: A Randomized, Double Blind, Placebo-controlled, Multicenter Study
NCT03172403	Terminated	Skeletal Muscle Expression of Myostatin and Cancer of Digestive System Associated Cachexia (MYOCAC Study)
NCT03245658	Terminated	The Effect of Medical Cannabis Inpatients with Palliative Pancreatic Cancer
NCT01501396	Terminated	Treatment of Cancer Anorexia-cachexia Syndrome (CACS) With Mirtazapine and Megestrol Acetate
NCT00535015	Terminated	A Randomized, Double-Blind, Pilot Study Comparing the Safety and Efficacy of Betamarc Plus Chemotherapy to Chemotherapy Alone in the Prevention and Treatment of CACS in Patients with Advanced NSCLC

… Supplemental File 1 Continue

Identification Number	Status	Study Title
NCT01330823	Terminated	L-Carnitine in the Palliative Treatment of Advanced Pancreatic Cancer (CARPAN): A Prospective, Randomised, Placebo Controlled, Double Blinded, Multicentre Trial
NCT03720158	Terminated	Effect of Enteral Supplementation of Omega 3 Fatty Acids on the Quality of Life and Functionality of Patients with Head and Neck Squamous Cell Carcinoma Undergoing Radiotherapy
NCT01046383	Terminated	Effect of IMN1207 Versus Casein on, Weight Loss, Survival and Quality of Life in Non-small Cell Lung Cancer Patients During or Following Chemotherapy, Radiation or Surgery: A Multi-center Randomized, Double-blind Study - Phase III Trial
NCT01132547	Terminated	Prevention of Cancer/Treatment-Related Weight Loss in Children at High Nutritional Risk
NCT03253029	Terminated	Use of Branched-chain Amino Acids in Cancer Cachexia
NCT02072057	Terminated	The RUXexia Trial: An Open-label Phase II Trial of Ruxolitinib in the Treatment of Cachexia in Patients with Tumor-Associated Chronic Wasting Diseases.
NCT01505764	Terminated	The Role of Ghrelin in Cancer Cachexia
NCT02416570	Terminated	Is Clarithromycin a Potential Treatment for Cachexia in People with Lung Cancer?
NCT00851448	Terminated	Safety, Tolerance and Efficacy of an Oral Nutritional Supplement in Lung Cancer Patients. A Randomised, Double-blind, Controlled Pilot Study
NCT00625742	Terminated	An Exploratory Trial of a Multimodal Treatment Strategy for Cancer Cachexia
NCT02580422	Terminated	Pilot Project for Cardiopulmonary and Functional Evaluation in Patients with Pancreatic Cancer Associated Cachexia

… Supplemental File 1 Continue

Identification Number	Status	Study Title
NCT04001010	Terminated/ Withdrawn	Safety and Efficacy of PPP011-kit for Improving Physical Functioning and for Modulating CachexiaProgression in Patients with Advanced Cancer and Associated Cachexia: A Randomized, Double Blind, Placebo Controlled, Parallel Group Study
NCT01865903	Terminated/ Withdrawn	Nutritional Problems and Changes in Body Composition in Patients with Non-small Cell Lung Cancer; Incidence, Development and Impact on Quality of Life, Adverse Effects and Survival.
NCT00127387	Terminated/ Withdrawn	A Prospective, Randomized Pilot Study of Enbrel VS Placebo in Patients Receiving Radiation Therapy to Combat Fatigue and Cachexia
NCT02650570	Terminated/ Withdrawn	Mitochondrial Respiratory Function in Mammalian Skeletal Muscle: Metabolic Insights into Cancer and Burn Cachexia Through Comparative Physiology of Humans and Marine Mammals

medRxiv preprint doi: https://doi.org/10.1101/2025.03.14.25323917; this version posted June 25, 2025. The copyright holder for this preprint (which was not certified by peer review) is the author/funder, who has granted medRxiv a license to display the preprint in perpetuity.
It is made available under a CC-BY 4.0 International license .

Supplemental File 2 - Summary of search results by terms and status of the clinical trials

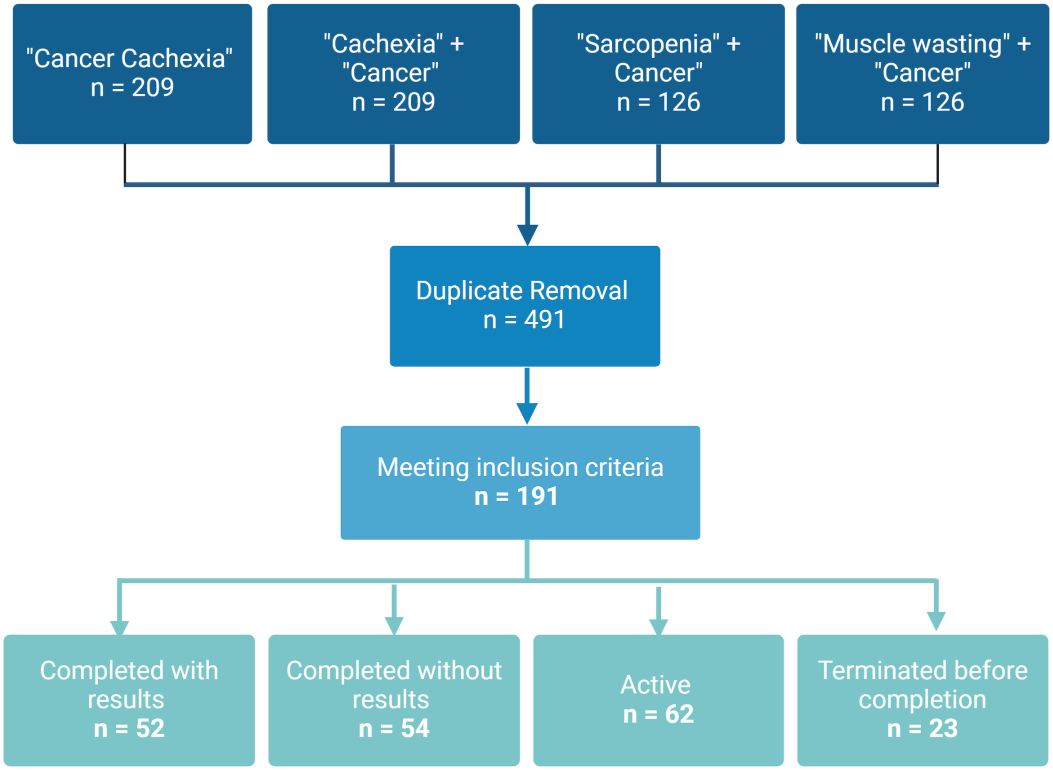

medRxiv preprint doi: https://doi.org/10.1101/2025.03.14.25323917; this version posted June 25, 2025. The copyright holder for this preprint (which was not certified by peer review) is the author/funder, who has granted medRxiv a license to display the preprint in perpetuity.
It is made available under a CC-BY 4.0 International license .

Supplemental File 3 - Reasons for withdrawn or anticipated termination before completion of clinical trials

Reason for Termination	Trials n (%)
Recruitment	10 (43.5)
Institutional Support & Staff Relocation	4 (17.4)
Funding	2 (8.7)
Change of Sponsor	1 (4.3)
Data variation	1 (4.3)
Time	1 (4.3)
Covid-19	1 (4.3)
Not Specified	3 (13.0)

medRxiv preprint doi: https://doi.org/10.1101/2025.03.14.25323917; this version posted June 25, 2025. The copyright holder for this preprint (which was not certified by peer review) is the author/funder, who has granted medRxiv a license to display the preprint in perpetuity.
It is made available under a CC-BY 4.0 International license .

Supplemental File 4 - Phases and status of clinical trials

Status/Phase	Phase 1	Phase 2	Phase 3	Phase 4	No Phase	Other*
**Completed With Results**	5	12	12	1	19	3
**Completed Without Results**	6	8	3	1	34	2
**Active**	4	5	0	0	52	1
**Withdrawn**	0	6	5	0	11	1
**Total**	15	31	20	2	116	7

Numbers represent the amount of clinical trials registered on ClinicalTrials.gov in Phase1-4 or No Phase and their status: completed with results, completed without results, active, or withdrawn. *One trial completed with results and two completed without results were in phase 1 and 2. Two trials completed with results, one active, and one withdrawn were in phase 2 and 3.

medRxiv preprint doi: https://doi.org/10.1101/2025.03.14.25323917; this version posted June 25, 2025. The copyright holder for this preprint (which was not certified by peer review) is the author/funder, who has granted medRxiv a license to display the preprint in perpetuity.
It is made available under a CC-BY 4.0 International license .

Supplemental File 5 - Clinical trials registered on ClinicalTrials.gov that used Fearon's definition or any other criteria to diagnose cancer cachexia within each status

Definition used	Completed with Results (n=52)		Completed Without Results (n=54)		Active (n=62)		Withdrawn (n=23)		Total (n=191)	
	n	%	n	%	n	%	n	%	n	%
Fearon	29	(55.8)	9	(16.6)	24	(38.7)	2	(8.7)	64	(33.5)
Other	14	(26.9)	19	(35.2)	8	(12.9)	13	(56.5)	54	(28.3)
Not Speficied	9	(17.3)	26	(48.2)	30	(48.4)	8	(34.8)	73	(38.2)

n represents the amount of clinical trials registered on ClinicalTrials.gov that used Fearon's definition or any other criteria to diagnose cancer cachexia. % refers to the percentage of clinical trials relative the total of clinical trials within each status (completed with results, completed without results, active, or withdrawn). The % in the "Total" column shows the percentage relative to the total of trials included in the study.

medRxiv preprint doi: https://doi.org/10.1101/2025.03.14.25323917; this version posted June 25, 2025. The copyright holder for this preprint (which was not certified by peer review) is the author/funder, who has granted medRxiv a license to display the preprint in perpetuity.
It is made available under a CC-BY 4.0 International license .

Supplemental File 6 - Clinical trials stratified by clinical trial status studying cachexia but classified under other terms different than "cachexia"

Other Terms Used for Trial Classification	Completed with Results (n=52)		Completed Without Results (n=54)		Active (n=62)		Withdrawn (n=23)		Total (n=191)	
	n	%	n	%	n	%	n	%	n	%
Sarcopenia	5	(9.6)	6	(11.1)	16	(25.8)	0	(0.0)	27	(14.1)
Weight loss	9	(17.3)	10	(18.5)	1	(1.6)	3	(13.0)	23	(12.0)
Muscle loss	4	(7.7)	6	(11.1)	9	(14.5)	1	(4.4)	20	(10.5)
Malnutrition	4	(7.7)	3	(5.5)	1	(1.6)	1	(4.4)	9	(4.7)

n represents the number trials classified as sarcopenia, weight loss, muscle loss, or malnutrition studies. Trials are stratified by clinical trial status (completed with results, completed without results, active, or withdrawn).

medRxiv preprint doi: https://doi.org/10.1101/2025.03.14.25323917; this version posted June 25, 2025. The copyright holder for this preprint (which was not certified by peer review) is the author/funder, who has granted medRxiv a license to display the preprint in perpetuity.
It is made available under a CC-BY 4.0 International license .

Supplemental File 7 - Study population of clinical trials registered on ClinicalTrials.gov investigating cancer cachexia

A)

Study Population		Completed Trials with Results (n=52) n (%)	Completed Trials Without Results (n=54) n (%)	Active Trials (n=62) n (%)	Withdrawn Trials (n=23) n (%)	Total (n=191) n (%)
Enrollment	1-50	22 (42.3)	19 (35.2)	18 (29.0)	13 (56.5)	72 (37.7)
	51-100	12 (23.1)	17 (31.5)	18 (29.0)	2 (8.7)	49 (25.7)
	101-500	15 (28.8)	13 (24.1)	22 (35.5)	1 (4.4)	51 (26.7)
	501-1000	2 (3.8)	2 (3.7)	2 (3.2)	0 (0.0)	6 (3.1)
	>1000	1 (1.9)	1 (1.9)	2 (3.2)	0 (0.0)	4 (2.1)
	Not specified	0 (0.0)	2 (3.7)	0 (0.0)	7 (30.4)	9 (4.7)

n represents the number of trials by range of participants enrolled to the clinical trials by clinical trial status (completed with results, completed without results, active, or withdrawn). % refers to the percentage of clinical trials relative the total of clinical trials within each status. The % in the "Total" column shows the percentage relative to the total of trials included in the study.

B)

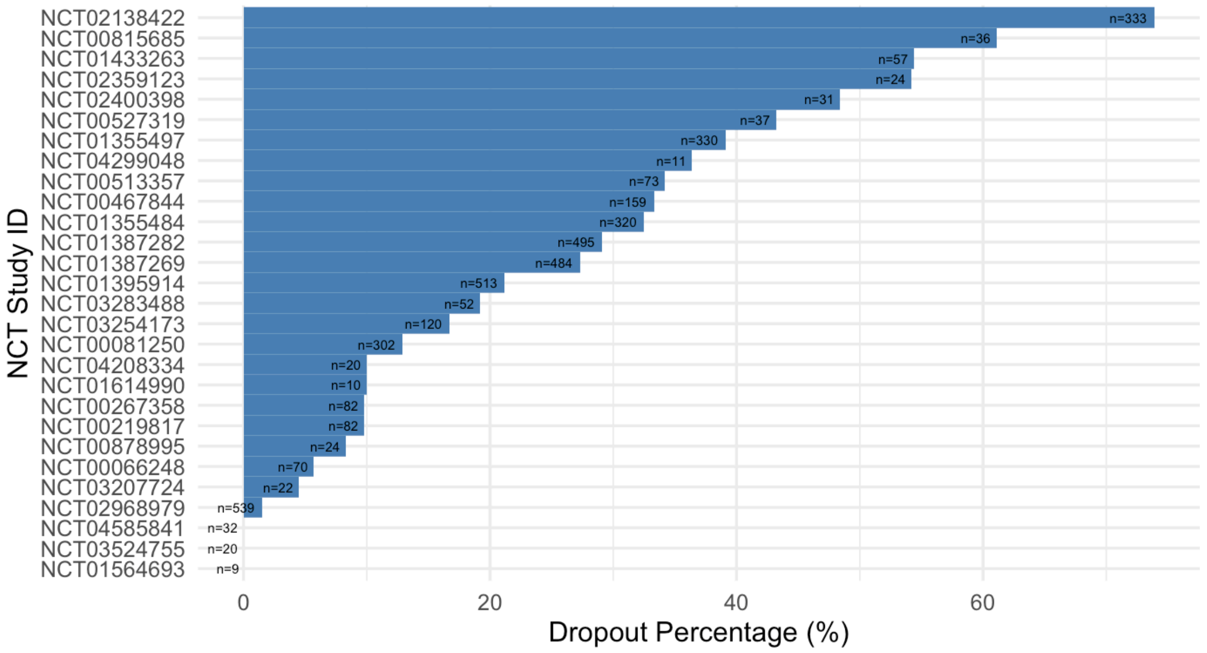

Dropout percentage (%) in the CTx completed with results that reported "Started" and "Not completed" populations. The dropout percentage is relative to each trial's population (n=28). The "Started" population is labeled on each bar.

medRxiv preprint doi: https://doi.org/10.1101/2025.03.14.25323917; this version posted June 25, 2025. The copyright holder for this preprint (which was not certified by peer review) is the author/funder, who has granted medRxiv a license to display the preprint in perpetuity.
It is made available under a CC-BY 4.0 International license .

Supplemental File 8 - Cancer type and cancer stage of clinical trials registered on ClinicalTrials.gov investigating cancer cachexia

Cancer Type and Stage		Completed Trials with Results (n=52)	Completed Trials Without Results (n=54)	Active Trials (n=62)	Withdrawn Trials (n=23)	Total (n=191)
		n (%)	n (%)	n (%)	n (%)	n (%)
**Type of Cancer Studied**	**Single cancer type**					
	Lung	8 (15.4)	6 (11.1)	10 (16.1)	5 (21.7)	29 (15.2)
	Pancreatic	3 (5.8)	4 (7.4)	11 (17.7)	4 (17.4)	22 (11.5)
	Gastric/GI*	3 (5.8)	5 (9.3)	8 (12.9)	1 (4.3)	17 (8.9)
	Colorectal	1 (1.9)	4 (7.4)	5 (8.1)	0 (0.0)	10 (5.2)
	Head & Neck	3 (5.8)	1 (1.9)	1 (1.6)	2 (8.7)	7 (3.7)
	Others	2 (3.8)	4 (7.4)	5 (8.1)	0 (0.0)	11 (5.8)
	**Combination of 2**					
	Lung + other	5 (9.6)	5 (9.3)	2 (3.2)	2 (8.7)	14 (7.3)
	GI + other	0 (0.0)	2 (3.7)	1 (1.6)	0 (0.0)	3 (1.6)
	**Combination of 3**					
	Lung + Pancreatic + Colorectal	1 (1.9)	0 (0.0)	2 (3.2)	1 (4.3)	4 (2.1)
	Others	1 (1.9)	2 (3.7)	2 (3.2)	1 (4.3)	6 (3.1)
	**Combination of 4 or more**	8 (15.4)	6 (11.1)	6 (9.7)	2 (8.7)	22 (11.5)
	**Not specified**	17 (32.7)	15 (27.8)	9 (14.5)	5 (21.7)	46 (24.1)
**Cancer Stage**	**Single stage**	0 (0.0)	2 (3.7)	2 (3.2)	2 (8.7)	6 (3.1)
	**Mixed**					
	Stage I-III	0 (0.0)	0 (0.0)	1 (1.6)	0 (0.0)	1 (0.5)
	Stage II-III	2 (3.8)	0 (0.0)	2 (3.2)	0 (0.0)	4 (2.1)
	Stage I-IV	0 (0.0)	1 (1.9)	1 (1.6)	1 (4.3)	3 (1.6)
	Stage II-IV	1 (1.9)	2 (3.7)	3 (4.8)	1 (4.3)	7 (3.7)
	Stage III-IV	9 (17.3)	5 (9.3)	4 (6.5)	6 (26.1)	24 (12.6)
	**Not Specified**	40 (76.9)	44 (81.5)	49 (79.0)	13 (56.5)	146 (76.4)

n represents the number of clinical trials that stated 1, 2, 3, or 4 and more cancer types and the cancer stage. % refers to the percentage of clinical trials relative the total of clinical trials within each status. The % in the "Total" column shows the percentage relative to the total of trials included in the study. *GI = gastrointestinal. Due to the uncertainty in some studies that reported gastric or gastrointestinal cancer without further details, gastrointestinal cancer was considered as a singular type of cancer rather than as a collection of conditions that can affect the entire gastrointestinal tract.

medRxiv preprint doi: https://doi.org/10.1101/2025.03.14.25323917; this version posted June 25, 2025. The copyright holder for this preprint (which was not certified by peer review) is the author/funder, who has granted medRxiv a license to display the preprint in perpetuity.
It is made available under a CC-BY 4.0 International license .

Supplemental File 9 - Clinical trials approach and intervention utilized of clinical trials registered on ClinicalTrials.gov investigating cancer cachexia

	Approach	Completed with Results (n=52)		Completed Without Results (n=54)		Active (n=62)		Withdrawn (n=23)		Total (n=191)	
		n	%	n	%	n	%	n	%	n	%
Study Type	**Observational**	7	(13.5)	20	(37.0)	20	(32.3)	4	(17.4)	51	(26.7)
	**Interventional**	45	(86.5)	34	(63.0)	42	(67.7)	19	(82.6)	140	(73.3)
Intervention	**Drug**	28	(53.8)	14	(25.9)	11	(17.7)	11	(47.8)	64	(33.5)
	**Dietary Supplement**	8	(15.4)	7	(13.0)	7	(11.3)	6	(26.0)	28	(14.7)
	**Exercise/Physical Therapy**	1	(1.9)	6	(11.1)	8	(12.9)	0	(0.0)	15	(7.9)
	**Multimodal**	6	(11.5)	3	(5.6)	8	(12.9)	1	(4.4)	18	(9.4)
	Dietary Supplement and Exercise	5	(9.6)	0	(0.0)	2	(3.2)	0	(0.0)	7	(3.7)
	Dietary Supplement and Drug	1	(1.9)	0	(0.0)	0	(0.0)	0	(0.0)	1	(0.5)
	Dietary Supplement and Nutrition Counseling	0	(0.0)	1	(1.9)	0	(0.0)	0	(0.0)	1	(0.5)
	Dietary Supplement, Exercise, and Drug	0	(0.0)	0	(0.0)	0	(0.0)	1	(4.4)	1	(0.5)
	Drug and Exercise	0	(0.0)	2	(3.7)	0	(0.0)	0	(0.0)	2	(1.1)
	Nutrition Counseling and Exercise	0	(0.0)	0	(0.0)	4	(6.5)	0	(0.0)	4	(2.1)
	Nutrition Counseling, Exercise, and Phycological	0	(0.0)	0	(0.0)	1	(1.6)	0	(0.0)	1	(0.5)
	Education and Psychological	0	(0.0)	0	(0.0)	1	(1.6)	0	(0.0)	1	(0.5)
	**Other**	2	(3.8)	4	(7.4)	10	(16.1)	1	(4.4)	17	(8.9)
	**None**	7	(13.5)	20	(37.0)	18	(29.0)	4	(17.4)	49	(25.7)

n represents the amount of clinical trials registered on ClinicalTrials.gov that utilized and observational or interventional approach for their study. This table also shows the intervention utilized (drug testing, dietary supplement, exercise/physical therapy, a combination of 2 or more interventions (multimodal), others, or none or non-stated intervention. % refers to the percentage of clinical trials relative the total of clinical trials within each status (completed with results, completed without results, active, or withdrawn). The % in the “Total” column shows the percentage relative to the total of trials included in the study.

medRxiv preprint doi: https://doi.org/10.1101/2025.03.14.25323917; this version posted June 25, 2025. The copyright holder for this preprint
(which was not certified by peer review) is the author/funder, who has granted medRxiv a license to display the preprint in perpetuity.
It is made available under a CC-BY 4.0 International license .

Supplemental File 10 - Pharmaceutical drugs and dietary supplements evaluated in CTx between 1995-2024.

CTx Identification Number	Treatment	Class
NCT00866970	ALD518	IL-6 inhibitor / Monoclonal antibody
NCT02138422, NCT03207724	Xilonix	Anti-IL-1α monoclonal antibody
NCT01395914, NCT01387282, NCT01387269, NCT03743064, NCT03637816, NCT01505764, NCT04844970, NCT00219817, NCT00267358, NCT00378131	Anamorelin HCl / RC-1291	Ghrelin receptor agonist / Appetite stimulant
NCT00066248, NCT01132547	Cyproheptadine hydrochloride	Antihistamine / Appetite stimulant
NCT00933361	Ghrelin	Peptide hormone (appetite stimulant)
NCT01614990	Macimorelin	Ghrelin receptor agonist
NCT03283488, NCT00004912, NCT00637728, NCT01501396, NCT00066248	Megestrol	Progestin / Appetite stimulant
NCT00625742	Atenolol	β-blocker (antihypertensive)
NCT01238107	MT-102	β-blocker + anabolic agent
NCT00895726	APD209	β-Adrenoceptor agonist
NCT00535015	Betamarc	β-adrenergic agent
NCT04815551, NCT05865535	AV-380	Anti-GDF-15
NCT05546476, NCT04299048, NCT04803305	Ponsegromab / PF-06946860	Anti-GDF15
NCT01433263	BYM338 / Bimagrumab	Anti-ActRII
NCT02359123	Cannabics	Cannabis-based therapy
NCT04585841	Cannabidiol (CBD)	Phytocannabinoid
NCT03245658	THC and CBD Mixture	Phytocannabinoids
NCT04001010	PPP011	Cannabinoid-based
NCT06166134, NCT06137508, NCT05954117, NCT00040885, NCT00060502, NCT01127386	Antineoplastic agents	Chemotherapy / Immunomodulators
NCT04600154	MS-20	Targeted cancer therapy
NCT02416570	Clarithromycin	Antibiotic
NCT00127387, NCT00046904	Enbrel / Etanercept	TNF inhibitor
NCT00040885, NCT00060502	Infliximab	TNF inhibitor
NCT00329615	Insulatard, flexpen	Long-acting insulin
NCT05919147	Pioglitazone	Thiazolidinedione (insulin sensitizer)
NCT03254173, NCT03283488	Mirtazapine / Remeron	Antidepressant / Appetite stimulant
NCT00489593	Olanzapine	Antipsychotic
NCT03263520	Nandrolone Decanoate; Dexamethasone	Anabolic steroid + Corticosteroid
NCT00878995	Testosterone Enanthate	Anabolic androgenic steroid
NCT01355497, NCT01355484, NCT00467844	GTx-024 / Enobosarm	Selective androgen receptor modulator (SARM)
NCT04906746, NCT02072057	Ruxolitinib	JAK1/2 inhibitor

medRxiv preprint doi: https://doi.org/10.1101/2025.03.14.25323917; this version posted June 25, 2025. The copyright holder for this preprint (which was not certified by peer review) is the author/funder, who has granted medRxiv a license to display the preprint in perpetuity.
It is made available under a CC-BY 4.0 International license .

… Supplemental File 10 Continue

CTx Identification Number	Treatment	Class
NCT04034745	Telotristat ethyl	Tryptophan hydroxylase inhibitor
NCT00527319	VT-122	COX-2 inhibitor + PPARγ agonist
NCT05336266	Ketorolac Tromethamine	Non-steroidal anti-inflammatory
NCT00196885	N-acetylcysteine	Antioxidant / Glutathione precursor
NCT00513357, NCT00625742	Melatonin	Hormone / Sleep aid / Antioxidant
NCT04208334, NCT05856500	Curcumin	Anti-inflammatory / Antioxidant
NCT00815685, NCT00031707	Eicosapentaenoic Acid	ω-3 fatty acid / Anti-inflammatory
NCT00094562, NCT04699760, NCT04161794	Fish oil supplement	ω -3 fatty acid / Anti-inflammatory
NCT01596933	BioMega SDA®	ω-3 fatty acid / Anti-inflammatory
NCT03720158, NCT06015971, NCT02779868, NCT00003077	Omega 3	ω-3 fatty acid / Anti-inflammatory
NCT05623852	Fucoidan	Immune-modulatory / Antioxidant
NCT00014248	Adenosine triphosphate	Cellular energy molecule
NCT00081250, NCT05856500	Creatine monohydrate	Ergogenic aid
NCT02561143	Atta	Nutritional supplement
NCT00625742, NCT00053053	Juven	Nutritional supplement
NCT02515032	Nutrifriend Cachexia	Nutritional supplement
NCT03316157	Prosure	Nutritional supplement
NCT04131426	Remune	Nutritional supplement
NCT04643613	Protison cancer nutritional formula (PCNF)	Nutritional supplement
NCT02877966	Amixea	Nutritional supplement
NCT05771207	Protein	Protein supplement
NCT01046383	IMN1207	Cysteine-Rich Non-Denatured Whey Protein Isolate
NCT01694602	3-methylhistidine (D-3MH)	Amino acid derivative
NCT01330823	L-Carnitine	Amino acid derivative
NCT06027242, NCT05023499	glutamine	Amino acid
NCT03253029	Branched Chain Amino Acid	Amino acid
NCT04098237	Pancrelipase	Digestive enzyme
NCT03144128, NCT04864431	Vitamin D	Vitamin
NCT05194397	Nicotinamide Riboside	Vitamin
NCT05264038	OC514	"Anticachexia agent" mechanism of action as of yet undisclosed

medRxiv preprint doi: https://doi.org/10.1101/2025.03.14.25323917; this version posted June 25, 2025. The copyright holder for this preprint (which was not certified by peer review) is the author/funder, who has granted medRxiv a license to display the preprint in perpetuity.
It is made available under a CC-BY 4.0 International license .

Supplemental File 11 – Biomarkers measured in cancer cachexia clinical trials registered on ClinicalTrials.gov

Category	Markers
Inflammation	CRP, TNF-a, TNF-b, IL-1, IL-1B, IL-2, IL-4, IL-5, IL-6, IL-7, IL-8, IL-10, IL-12, IL-13, IL-15, IL-1ra, IFN Gamma, neutrophil/lymphocyte ratio, activin A, Myostatin, MCP1, ICAM-1, bFGF, G-CSF, Eotaxin, CCL11, IP10, CXCL10, PDGF, VEGF, RANKL, OPG, DLL1, Periostin, CCL5, CCL4, MIP1b, CCL2, CCL3, IGF1, IGFBP3, GDF11, GDF15, p38, IKK, p65, ERK1/2, JNK, p-selectin.
Metabolism	Blood glucose, Glucose, Proteasome Activity, Serum Albumin, Pre-albumin, Albumin, Total protein, Fibrinogen, Calcium, Sodium, Chloride, Bicarbonate, LDH, Triglycerides, Glycerol, Free fatty acids, Cholesterol, Transferrin, Hb1Ac, Vitamin A, Vitamin E.
Hematology	Hemoglobin, Hematocrit, Erythrocytes, Mean Corpuscular Volume, Leukocytes, Lymphocytes, Platelets, Coagulation test, NK cells, T cells.
Liver/Kidney Function	Bilirubin, Aspartate Aminotransferase, Alkaline Phosphatase, Creatinine, ALT, AST, Urea, Protein.
Urianalysis	Urine Glucose, Ketones, Urine Protein, Urine Hemoglobin, Urobilinogen, Nitrite, Leukocyte Esterase, Hyaline Casts, Urinary 3-methylhistidine.
Homones	Leptin, Ghrelin, Insulin, TSH, LH, FSH, E2, DHEA-S, Testosterone, T4, GLP1, NPY.
Molecular/genetic	EGFR, ALK, ROS1, BRAF, HER2, K-RAS, UBE2B, UBE2D2, MaFbx, MuRF1, Nedd4, Mdm2, 26S proteasome subunits, beclin, LAMP, PARP, K48, K63, NRF2, KEAP1, HSP, ER-stress, Pax3, Pax7, Myf5, M-cadherin, myogenin, desmin, SMAD3.
Microbiome	Stool sample microbiome analysis, Fecal microbiota, Gut permeability markers, Microbial metabolites.
Muscle metabolism/structure	Muscle biopsies, Muscle protein synthesis, ATP concentration in myotubes, Mitochondrial respiration.
Bone metabolism/structure	RANKL, Osteoprotegerin, Vitamin D, Calcium, Phosphate, Vitamin K, vitamin K-PT.
Biopsies	Adipose tissue biopsy, Muscle biopsy, Tumour biopsy, Subcutaneous adipose tissue, Histological analysis.
Others	Total RNA, Reactive oxygen species, Apoptosis markers, Caspase-3, Phosphorylated Akt, Nitrosylated HNE, ATG.

medRxiv preprint doi: https://doi.org/10.1101/2025.03.14.25323917; this version posted June 25, 2025. The copyright holder for this preprint
(which was not certified by peer review) is the author/funder, who has granted medRxiv a license to display the preprint in perpetuity.
It is made available under a CC-BY 4.0 International license .

Supplemental File 12 - Outcome measures of cachexia in the clinical trials registered on ClinicalTrials.gov by status

Measures of Cachexia		Completed with Results (n=52)		Completed Without Results (n=54)		Active (n=62)		Withdrawn (n=23)		Total (n=191)	
		n	%	n	%	n	%	n	%	n	%
Outcome Measures	**Body weight**	26	(50.0)	20	(37.0)	24	(38.7)	11	(47.8)	81	(42.4)
	**Body composition**	34	(65.4)	26	(48.1)	45	(72.6)	14	(60.9)	119	(62.3)
	Muscle mass	8	(15.4)	11	(20.4)	29	(46.8)	1	(4.3)	49	(25.7)
	Lean body mass	19	(36.5)	10	(18.5)	10	(16.1)	6	(26.1)	45	(23.6)
	Fat mass	7	(13.5)	7	(13.0)	7	(11.3)	0	(0.0)	21	(11.0)
	**Physical function**	31	(59.6)	22	(40.7)	30	(48.4)	13	(56.5)	96	(50.3)
	Muscle function	22	(42.3)	9	(16.7)	15	(24.2)	10	(43.5)	56	(29.3)
	Physical activity levels	8	(15.4)	4	(7.4)	13	(21.0)	1	(4.3)	26	(13.6)
	Fatigue	8	(15.4)	3	(5.6)	2	(3.2)	1	(4.3)	14	(7.3)
	**Quality of life (QoL)**	23	(44.2)	10	(18.5)	25	(40.3)	13	(56.5)	71	(37.2)
	**Nutrition status/appetite**	22	(42.3)	10	(18.5)	15	(24.2)	11	(47.8)	58	(30.4)
	**Molecular**	19	(36.5)	21	(38.9)	38	(61.3)	9	(39.1)	87	(45.5)
	**Safety & tolerability**	13	(25.0)	11	(20.4)	10	(16.1)	7	(30.4)	41	(21.5)
	**Survival**	10	(19.2)	11	(20.4)	12	(19.4)	4	(17.4)	37	(19.4)

n represents the number of outcomes stated during the clinical trials (body weight, body composition, physical function, quality of life, nutrition status/appetite, molecular, safety and tolerability, survival, or others). % refers to the percentage of clinical trials relative the total of clinical trials within each status (completed with results, completed without results, active, or withdrawn). The % in the "Total" column shows the percentage relative to the total of trials included in the study.

medRxiv preprint doi: https://doi.org/10.1101/2025.03.14.25323917; this version posted June 25, 2025. The copyright holder for this preprint (which was not certified by peer review) is the author/funder, who has granted medRxiv a license to display the preprint in perpetuity.
It is made available under a CC-BY 4.0 International license .

Supplemental File 13 - Measurement tools used to investigate cachexia

	Measures of Cachexia	Completed with Results (n=52)		Completed Without Results (n=54)		Active (n=62)		Withdrawn (n=23)		Total (n=191)	
		n	%	n	%	n	%	n	%	n	%
Measurement Tools	**Body Composition Assessment**										
	CT	7	(13.5)	7	(13.0)	19	(30.6)	2	(8.7)	35	(18.3)
	BIA	12	(23.1)	8	(14.8)	8	(12.9)	5	(21.7)	33	(17.3)
	DXA	8	(15.4)	3	(5.6)	8	(12.9)	5	(21.7)	24	(12.6)
	MRI	2	(3.8)	2	(3.7)	4	(6.5)	1	(4.3)	9	(4.7)
	Skinfold	4	(7.7)	0	(0.0)	1	(1.6)	3	(13.0)	8	(4.2)
	MUAC	3	(5.8)	1	(1.9)	0	(0.0)	1	(4.3)	5	(2.6)
	PET	0	(0.0)	1	(1.9)	3	(4.8)	0	(0.0)	4	(2.1)
	Waist-Hip ratio	0	(0.0)	1	(1.9)	2	(3.2)	0	(0.0)	3	(1.6)
	**Physical Function Assessment**										
	Hand Grip Strenght	19	(36.5)	7	(13.0)	15	(24.2)	9	(39.1)	50	(26.2)
	Walk test	5	(9.6)	6	(14.8)	13	(21.0)	1	(4.3)	25	(13.1)
	Sit-to-stand	3	(5.8)	3	(5.6)	9	(14.5)	3	(13.0)	18	(9.4)
	Stair Climb	5	(9.6)	2	(3.7)	3	(4.8)	2	(8.7)	12	(6.3)
	SPPB	0	(0.0)	5	(9.3)	3	(4.8)	0	(0.0)	8	(4.2)
	Accelorometry	3	(5.8)	0	(0.0)	1	(1.6)	0	(0.0)	4	(2.1)
	**Symptom and QoL Assessment**										
	ECOG	23	(44.2)	12	(22.2)	19	(30.6)	9	(39.1)	63	(33.0)
	EORTC QLQ	12	(23.1)	8	(14.8)	13	(21.0)	7	(30.4)	40	(20.9)
	FAACT	11	(21.2)	7	(13.0)	7	(11.3)	1	(4.3)	26	(13.6)
	Karnofsky scale	5	(9.6)	3	(5.6)	4	(6.5)	5	(21.7)	17	(8.9)
	FACIT-F	4	(7.7)	2	(3.7)	2	(3.2)	0	(0.0)	8	(4.2)
	**Nutrition and Appetite Assessment**										
	Dietary recall/food diary	4	(7.7)	5	(9.3)	5	(8.1)	4	(17.4)	18	(9.4)
	PG- SGA	2	(3.8)	1	(1.9)	2	(3.2)	2	(8.7)	7	(3.7)
	VAS	4	(7.7)	0	(0.0)	0	(0.0)	2	(8.7)	6	(3.1)
	SNAQ	2	(3.8)	1	(1.9)	1	(1.6)	0	(0.0)	4	(2.1)
	**Molecular Assessment**										
	Blood sample	17	(32.7)	25	(46.3)	34	(54.8)	9	(39.1)	85	(44.5)
	Muscle biopsy	0	(0.0)	7	(13.0)	11	(17.7)	3	(13.0)	21	(11.0)

CT: Computed Tomography, BIA: Bioelectrical Impedance Analysis, DXA: Dual Energy X-ray Absorptiometry, MRI: Magnetic Resonance Imaging, MUAC: Mid-Upper Arm Circumference, PET: Positron Emission Tomography, SPPB: Short Physical Performance Battery, ECOG: Eastern Cooperative Oncology Group, EORTC QLQ: Research and Treatment of Cancer Quality of Life (QoL) Questionary, FAACT: Functional Assessment of Anorexia/Cachexia Treatment, FACIT-F: Functional Assessment of Chronic Illness Therapy – Fatigue, PG- SGA: Patient-Generated Subjective Global Assessment, VAS: Visual Analog Scale, SNAQ: Simplified Nutritional Appetite Questionnaire.
